# Advances in the Study of Endometrial Receptivity and Glucose Metabolism Mechanisms in Insulin Resistance

**DOI:** 10.1002/edm2.70249

**Published:** 2026-06-11

**Authors:** Si‐yan Huang, Shao‐rong Chen, Wei‐hong Chen, Ling‐tao Zheng, Jing Xu, Yan‐Chuan Shi, Shu Lin, Qi‐rong Shi

**Affiliations:** ^1^ Department of Gynaecology and Obstetrics The Second Affiliated Hospital of Fujian Medical University Quanzhou Fujian China; ^2^ Group of Neuroendocrinology Garvan Institute of Medical Research Sydney Australia; ^3^ Centre of Neurological and Metabolic Research The Second Affiliated Hospital of Fujian Medical University Quanzhou Fujian China

**Keywords:** endometrial glucose metabolism, endometrial receptivity, insulin resistance

## Abstract

**Introduction:**

Insulin resistance (IR) is one of the important pathologic bases of polycystic ovary syndrome (PCOS) and an important factor contributing to infertility. In patients with PCOS, IR acts not only on insulin‐sensitive tissues, such as skeletal muscle, adipose tissue, and liver, but also on the ovaries and endometrium. Localised endometrial IR is closely associated with impaired endometrial receptivity, and proper glucose metabolism is a key factor for embryo implantation.

**Methods:**

This paper describes the signalling pathways of endometrial glucose metabolism and the transcription factors regulated by endometrial glucose metabolism signalling pathways.

**Results:**

The purpose of this paper is to explore the mechanism of endometrial glucose uptake in local IR and find key intersections in this mechanism. For example, Foxo1, as the intersection of the Insulin Receptor Substrate‐Phosphatidylinositol3‐kinase‐Protein Kinase B‐Glucose Transporters (IRS‐PI3K‐Akt‐GLUTs) pathway and the Cyclic Adenosine Monophosphate‐ Protein Kinase A‐ Calcium ion (cAMP‐PKA‐Ca^2+^) pathway, and as an important biomarker for evaluating endometrial receptivity. It is of great significance for studying the improvement of endometrial receptivity.

**Conclusions:**

This article summarises the therapeutic approaches targeting insulin resistance and the enhancement of endometrial receptivity based on the above pathways, so as to provide references and suggestions for future therapeutic strategies.

## Graphical abstract

This article systematically reviews how insulin resistance (IR) disrupts endometrial receptivity by interfering with glucose metabolism mechanisms, ultimately leading to infertility in patients with Polycystic Ovary Syndrome (PCOS).

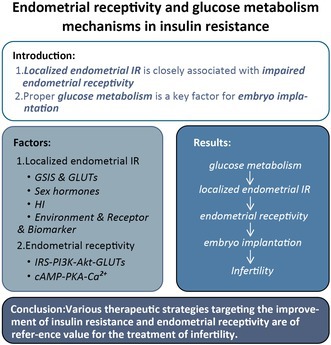

**Abbreviations**
3D PDUS: three‐dimensional power Doppler ultrasound
Akt: protein Kinase B (PKB)
AMH: anti‐Müllerian hormone
AMPK: adenosine 5′‐monophosphate (AMP)‐activated protein kinase
AR: androgen receptor
AS160: Akt 160 kDa substrate
BMP2: bone morphogenetic protein2
Ca^2+^
: calcium ion
cAMP: cyclic adenosine monophosphate
CYP19A1: cytochrome P450 family 19 subfamily A Member 1
EMT: epithelial‐mesenchymal transition
ER: oestrogen receptor
ERK: extracellular regulated protein kinases
FFA: free fatty acids
Foxo1: Forkhead Box O1
FSH: follicle‐stimulating hormone
FSHR: Follicle‐stimulating hormone receptor
GLP‐1: glucagon‐like peptide‐1
GLUT: glucose transporter
GPER: G protein‐coupled Oestrogen Receptor
GSIS: glucose‐stimulated insulin secretion
hESC: human embryonic stem cell
HI: hyperinsulinaemia
HIF‐1α: hypoxia inducible factor 1alpha
HOXA10: homeobox A10
IGFBP‐7: insulin‐like growth factor binding protein 7
IR: insulin resistance
IRS: insulin receptor substrate
IVF: in vitro fertilisation
LH: luteinising hormone
LHCGR: luteinising hormone/choriogonadotropin receptor
LPAR3: lysophosphatidic acid receptor 3
MAPK: mitogen‐activated protein kinase
MEF‐2A: myocyte enhancer factor 2A
MSCs: mesenchymal stem cells
mTOR: mammalian target of rapamycin
NR4A1: nuclear receptor 4 A1
NR4A2: nuclear receptor 4 A2
OS: oxidative stress
OXPHOS: oxidative phosphorylation
PCOS: polycystic ovary syndrome
PGRMC1: progesterone receptor membrane component 1
PI3K: phosphatidylinositol 3‐kinase
PKA: protein kinase A
p‐mTOR: phosphorylated mTOR
PR: progesterone receptor
PRL: prolactin
PYGB: glycogen phosphorylase B
RIF: recurrent implantation failure
ROS: reactive oxygen species
SIK2: salt‐inducible kinase 2
TBC1D4: TBC1 domain family member 4
TCA: tricarboxylic acid
TCM: traditional Chinese medicine
VEGFA: vascular endothelial growth factor A

## 1 Introduction

Insulin resistance (IR) has been widely recognised as one of the heterogeneous clinical manifestations of polycystic ovary syndrome (PCOS), as demonstrated by numerous studies [1]. It is closely associated with other clinical features of PCOS, particularly hyperinsulinaemia (HI). Both IR and HI are considered fundamental contributors to the pathophysiology of PCOS and play critical roles in the development of reproductive dysfunction through various mechanisms [1, 2]. However, the precise relationship between IR and HI remains controversial. Some studies suggest that HI is a compensatory response to IR, while others propose that HI may precede and potentially induce IR. Despite these differing viewpoints, the strong association between IR and HI is widely acknowledged.

In the reproductive system, the endometrium, which contains insulin receptors, can develop IR, thereby disrupting local glucose homeostasis [3, 4]. Under conditions of IR, GLUTs on the cell membrane fail to respond adequately, impairing glucose uptake into cells. This results in hyperglycaemia, which in turn contributes to the development of HI through compensatory mechanisms [5]. Insulin also plays a crucial regulatory role in the HPO axis. By activating insulin receptors in the pituitary, it modulates gonadotropin secretion and promotes androgen production in the ovaries [6]. Additionally, insulin interacts with FSH and LH signalling pathways in the ovary, thereby influencing ovarian function and directly modulating glucose metabolism in the endometrium [7].

Endometrial glucose metabolism is essential for maintaining endometrial receptivity, which is a prerequisite for successful embryo implantation. Adequate glucose utilisation ensures sufficient energy supply and supports the structural and functional integrity of the endometrium. Accumulating evidence indicates that IR impairs endometrial function and disrupts embryo implantation, ultimately contributing to infertility. In PCOS, IR is commonly associated with metabolic disturbances such as obesity, chronic inflammation, and hyperandrogenism. These factors collectively impair the translocation of GLUT‐4 to the cell membrane, thereby contributing to abnormal endometrial glucose metabolism [8]. Furthermore, IR and HI influence the local secretion of insulin‐like growth factors and their binding proteins within the endometrium, leading to dysregulated endometrial proliferation and functional deficiencies [9]. Inadequate glucose metabolism also compromises energy availability in endometrial tissue, further impairing the implantation process. These alterations result in a significant decline in endometrial receptivity. Proteomic and bioinformatic analyses of human endometrial tissue have suggested that local IR may represent a critical mechanism underlying impaired endometrial receptivity in individuals with PCOS [10].

Several signalling pathways have been identified as being associated with local endometrial IR, including the IRS–PI3K–Akt–GLUTs pathway, the mitogen‐activated protein kinase/extracellular signal‐regulated kinase (MAPK/ERK) pathway, the adenosine 5′‐monophosphate‐activated protein kinase/mammalian target of rapamycin (AMPK‐mTOR) pathway, and the FSH‐cAMP‐PKA‐Ca^2+^ pathway [11, 12, 13]. These pathways play distinct roles in regulating endometrial glycolysis and decidualisation, both of which are critical for establishing a receptive endometrium. In this review, we examine the physiological mechanisms governing endometrial glucose metabolism, as well as the pathological alterations associated with IR. We summarise the transcription factors that modulate these signalling cascades and influence endometrial receptivity. In addition, we discuss recent advances in therapeutic strategies targeting these mechanisms to improve endometrial function in the context of IR.

## 2 IR

### 2.1 A Brief Introduction of Glucose‐Stimulated Insulin Secretion in Reproductive System

#### 2.1.1 Steroid Hormones Play a Coordinated Part in Glucose‐Stimulated Insulin Secretion (GSIS)

**FIGURE 1:**
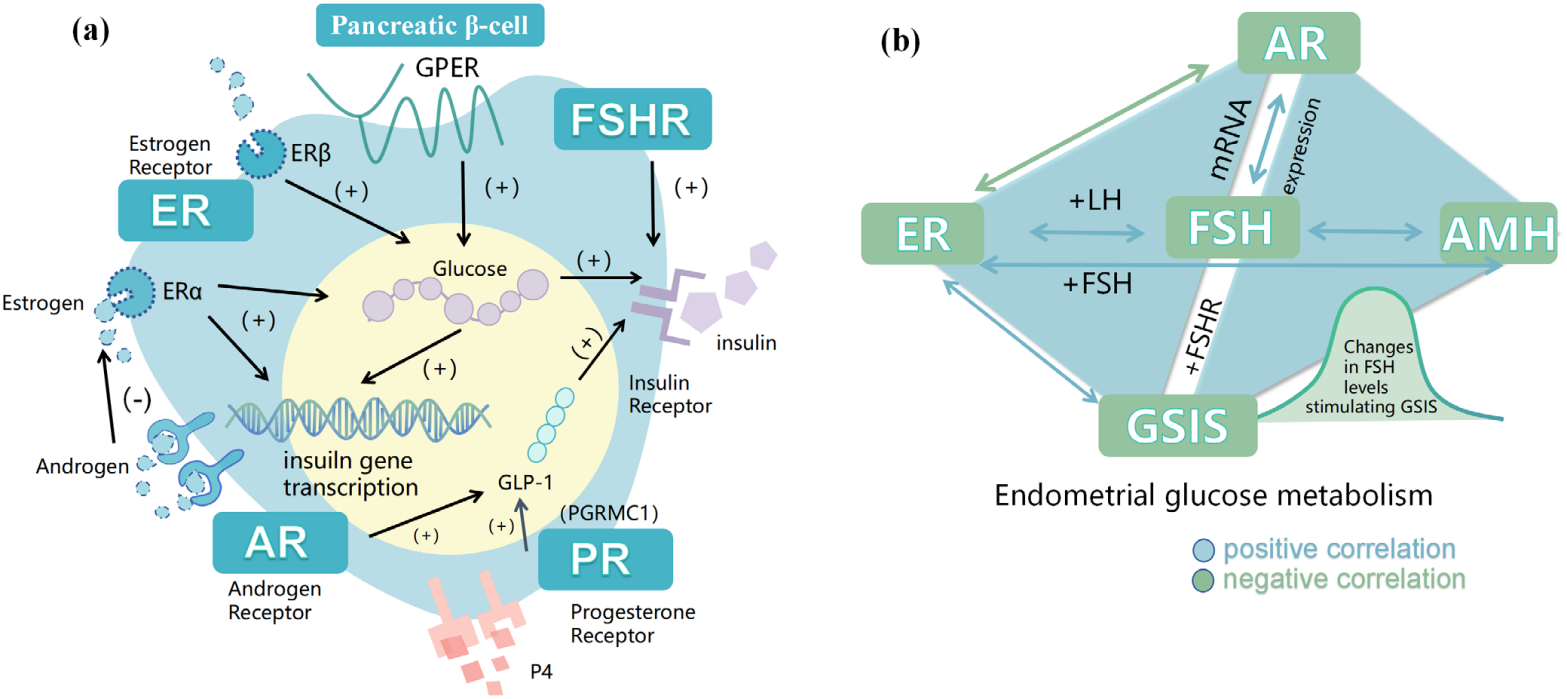
The roles of sex hormone receptors in endometrial and islet cells. This figure outlines insulin resistance mechanisms in the endometrium. (a) Pancreatic β‐cell insulin gene transcription is regulated by oestrogen (via ERα and ERβ), androgen (via AR), and progesterone (via PR). (b) Endometrial insulin resistance involves interactions of receptors and hormones (ER, AR, FSHR, LH, AMH) affecting glucose metabolism. The figure also shows pancreatic β‐cell and endometrial interactions, including insulin receptor and GSIS, which influence endometrial insulin resistance development.

The endometrium must attain a minimum threshold of energy reserves to initiate reproductive processes, making glucose metabolism and insulin secretion critical components of endometrial function. Hormones that regulate the endometrial growth cycle—oestrogen, androgen, and progesterone—are closely associated with insulin signalling, as their corresponding steroid receptors are also expressed in pancreatic β‐cells (Figure 1a). Among these, oestrogen receptors (ERs) play a particularly important role in modulating insulin secretion and have been widely studied for therapeutic applications. Specifically, ERα promotes insulin gene transcription and enhances glucose‐stimulated insulin secretion (GSIS), while ERβ and G protein‐coupled oestrogen receptor (GPER) also facilitate GSIS through distinct mechanisms [14, 15]. The androgen receptor (AR) directly influences both the endometrium and ovarian function. Experimental models have demonstrated that androgens can be used to induce PCOS‐like phenotypes in mice, highlighting their role in reproductive pathology. AR has also been shown to downregulate ER expression, thereby attenuating its regulatory effects on the endometrium and β‐cell function [16]. However, recent studies have identified AR as a positive modulator of GSIS, particularly by enhancing the insulinotropic action of glucagon‐like peptide‐1 (GLP‐1) [17]. Progesterone receptor membrane component 1 (PGRMC1), a membrane‐associated isoform of the progesterone receptor (PR), mediates non‐genomic P4 signalling and has been shown to facilitate GLP‐1‐induced insulin secretion. Additionally, islet β‐cells exhibit adaptive responses when PR antagonises the delayed proliferative effects of prolactin (PRL) [14, 18].

Recent studies have indicated that FSH plays a central role in regulating steroid hormone activity within the reproductive system. Its binding to FSH receptors (FSHR) on pancreatic β‐cells has been shown to influence insulin secretion. Notably, FSH‐induced GSIS follows a bell‐shaped dose–response curve. Both FSHR knockout mouse models and in vitro studies have demonstrated that disruption of FSH signalling results in impaired insulin secretion, subsequently leading to dysregulated glucose metabolism [19]. Beyond its metabolic implications, the primary physiological role of FSH is to regulate the female reproductive system through interactions with steroid hormones. For instance, FSHR expression is positively correlated with AR expression at both the protein and mRNA levels [20]. These interactions constitute a dual triangular regulatory network involving AMH, FSHR, ER, and AR, which collectively control endometrial proliferation and shedding (Figure 1b). Given these interconnections, mapping glucose metabolism pathways with FSH as a central regulatory node provides valuable insights and a promising direction for future research in reproductive and metabolic health [21].

#### 2.1.2 Glucose and Glucose Transporters in Endometrium

The translocation of GLUT4 represents a critical step in insulin‐stimulated glucose uptake and is mediated directly via the Akt‐TBC1 domain family member 4 (TBC1D4) signalling axis. Phosphoproteomic analyses have revealed that impaired GLUT4 translocation is a key signalling alteration observed in IR conditions [22]. As an insulin‐dependent glucose transporter, GLUT4 exhibits peak expression in the follicular phase of the menstrual cycle, whereas the insulin‐independent transporter GLUT1 is most abundantly expressed during the luteal phase [23]. Data feedback on whether GLUT 4 is expressed on the endometrium is inconsistent [24]. Despite this, numerous studies have reported that GLUT4 expression is significantly downregulated in the presence of hyperinsulinemia, while treatment with metformin has been shown to increase both mRNA and protein levels of GLUT4. Additionally, evidence suggests that steroid hormones may participate in the regulation of GLUT4 expression and activity [25]. GLUT1 was the first glucose transporter identified in the endometrium and is responsible for the transport of various hexoses, including glucose, mannose, galactose, glucosamine, and reduced ascorbic acid. In addition to its expression in the endometrium, GLUT1 is also highly expressed in the placenta and is considered the most abundantly transcribed glucose transporter in endometrial tissue. Its presence is essential for endometrial decidualisation and functional transformation during the menstrual cycle [23, 26, 27]. GLUT3, in contrast, shows elevated expression during menstruation and early pregnancy. Compared with GLUT1, GLUT3 expression declines significantly in the second trimester, suggesting its critical role in early embryonic development following implantation. For instance, progesterone has been shown to modulate GLUT3 expression via the upregulation of miR‐152, thereby inhibiting early embryo development and implantation through downregulation of GLUT3 in the endometrium [28].

#### 2.1.3 Characteristics of Glycolysis

As the primary metabolic pathway in the endometrium, glycolysis plays an essential role in energy metabolism during the menstrual cycle and throughout pregnancy. Inhibition of glycolysis has been shown to reduce inflammation and impair endometrial repair during the menstrual phase. In mouse models, stable expression of hypoxia‐inducible factor 1 alpha (HIF‐1α) under hypoxic conditions was found to be associated with the inhibition of glycolysis rather than hypoxia‐induced disruption of endometrial repair, confirming the independent effect of glycolysis on endometrial function [29]. In addition, HIF‐1α has been shown to increase the expression of GLUT1 and multiple glycolytic enzymes, with expression levels peaking at 7 weeks of gestation and declining between 10 and 12 weeks. These dynamic changes in HIF‐1α expression indicate that glycolysis is functionally involved in early pregnancy. Moreover, glycolysis has been shown to promote the proliferation, migration, and invasion of trophoblast cells, and to influence the process of endometrial decidualisation. It is also required for the proliferation, secretion, and cytotoxic activity of decidual natural killer (dNK) cells [30].

### 2.2 IR and Related‐HI

#### 2.2.1 IR Effect the Insulin Secretion: The Dynamic Clustering of Insulin Receptor

As previously mentioned, the endometrium expresses insulin receptors, and prolonged exposure to elevated insulin levels leads to reduced insulin sensitivity and impaired insulin signalling, ultimately resulting in insulin resistance (IR). In IR cells, the ability of insulin receptors to aggregate into clusters under acute insulin stimulation is diminished, indicating altered receptor dynamics. However, metformin has been shown to restore insulin receptor dynamics by decreasing the levels of reactive oxygen species (ROS), highlighting its therapeutic value in the management of IR [4, 31].

#### 2.2.2 HI Is Often Both a Result and a Driver of Insulin Resistance

Several studies have confirmed that long‐term exposure to elevated insulin levels leads to decreased tyrosine and serine autophosphorylation of insulin receptors, a reduction that is not reversed even after insulin withdrawal. Recent findings have demonstrated that during the decidualisation of human endometrial stromal cells (hESC), Nuclear Receptor 4A1 (NR4A1) and Nuclear Receptor 4A2 (NR4A2) function as metabolic mediators of insulin signalling within the PI3K‐AKT pathway. This provides the first evidence that insulin interferes with the decidualisation process of hESC through the PI3K/AKT‐NR4A axis [32]. Whether due to insulin receptor dysfunction or interference with downstream insulin signalling pathways, the consequence is reduced tissue insulin sensitivity, ultimately leading to insulin resistance (IR). Hyperinsulinemia (HI) further contributes to the development of IR, creating a self‐reinforcing cycle. At the same time, HI is also a consequence of IR. In vivo studies involving sustained insulin infusion have confirmed that HI can induce IR. Specifically, persistent HI for 40 h significantly reduces glucose uptake and overall glucose metabolism, even when plasma insulin concentrations are below maximal or maximally effective levels [33].

## 3 Local Endometrial IR

### 3.1 The Introduction of Endometrial Receptivity

#### 3.1.1 Background

The concept of endometrial receptivity refers to the specific period within the menstrual cycle during which the endometrium becomes capable of receiving and supporting the implantation of a developing embryo. This process is dynamic and stringently regulated through a complex interplay of molecular, cellular, and hormonal mechanisms. The successful establishment of endometrial receptivity is essential for embryo implantation, and any disruption may lead to implantation failure or early pregnancy loss [34]. In recent years, considerable progress has been made in elucidating the underlying mechanisms of endometrial receptivity. The subsequent sections will summarise various influencing factors, including environmental cues, hormonal regulation, receptor signalling, and molecular biomarkers. However, effective diagnostic tools and therapeutic strategies remain limited. One of the recent research focuses has been the application of mesenchymal stem cells (MSCs) in the treatment of endometrial dysfunction. Owing to their multipotent differentiation capacity and immunomodulatory properties, in vivo studies have confirmed that MSCs can effectively increase endometrial thickness and improve endometrial receptivity after transplantation [35, 36].

#### 3.1.2 Influencing Factors (Table 1)

**TABLE 1:** Factors affecting endometrial receptivity and their corresponding mechanisms.

Category	Factor	Pathway of influence	References
Environment	Age	Cellular senescence. Increased pro‐inflammatory and tissue fibrosis. Affects epigenetic regulation of the endometrium. Impaired decidualisation of endometrial stromal cells.	[37, 38, 39]
	obesity	Fatty acid‐related pro‐inflammatory response mediated by prostaglandin signalling. Phospholipid‐derived mediators. Synergistically mediated by steroid hormones progesterone and oestrogen. Delayed WOI. Impairs endometrial decidualisation. Affects the activity and function of chemokines, cytokines and the immune system as well as structural extracellular matrix and protein‐binding molecules. Changes the abundance of endometrium‐specific proteins.	[40, 41, 42, 43, 44, 45, 46]
Receptor	IGFR	miR‐145 affects embryo attachment by reducing IGFR in the endometrium. miR‐140 inhibits embryo implantation by downregulating IGFR expression. The H19/Let‐7/IGF1R regulatory pathway affects the proliferation of endometrial cells.	[47, 48]
	ER	Affects endometrial implantation window. Participates in human endometrial stromal cell cycle regulation, progesterone response and stromal growth together with E2 activation.	[49, 50]
	PR	A genomic region that regulates the receptivity and key functional processes of the uterus. Involved in P4 signal transduction during embryo implantation.	[51, 52]
	AR	Differential expression of endometrial receptivity and decidualisation genes in early and mid‐pregnancy. Regulation of gene expression in decidualising stromal cells.	[53, 54]
Biomarker	integrin	Affects endometrial receptivity during high androgen states. Indirectly acts via endometrial fluid. Plays an important role in the trophoblast‐endometrial adhesion of embryo implantation. Down‐regulation of oestrogen and progesterone receptors is related to it. Integrin beta8 (ITGB8) promotes endometrial receptivity by activating the VAV‐RAC1 signalling axis through FAK.	[54, 55, 56]
	GLUT4	Expression increases during implantation window. Impairs endometrial receptivity by increasing the concentration of glucose in uterine fluid, inhibiting isoprene formation and the expression of leukaemia inhibitory factor (LIF) and integrin β3.	[9, 57]

1. Environment: age [37, 38, 39]/obesity [40, 41, 42, 43, 44, 45, 46]/inflammation
2. Receptor: insulin receptor/IGFR [47, 48]/ER [49, 50]/PR [51]/AR [53, 54].
3. Biomarker: integrin [55, 56, 58]/GLUT4 [9, 57]/pps/HOXA‐10/Foxo1.

### 3.2 The Character of Endometrial IR

#### 3.2.1 Affect the Expression of Endometrial Receptivity‐Related Molecules

Local endometrial insulin resistance (IR) is associated with hyperandrogenemia, obesity, and chronic low‐grade inflammation, particularly with imbalances in oxidative stress (OS), which contribute to reactive oxygen species (ROS)‐mediated OS elevation and pregnancy complications [59]. Endometrial IR primarily results from impaired key components of the local insulin signalling pathway and disrupted downstream transduction, ultimately reducing glucose uptake. These impairments include downregulation of glucose transporter 4 (GLUT‐4), decreased phosphorylation of insulin receptor substrates (INSR), and transcriptional inhibition of forkhead box O1 (FOXO1) in the endometrium. Transcription factors such as adenosine monophosphate‐activated protein kinase (AMPK) and myocyte enhancer factor 2A (MEF‐2A) have been identified as regulators of GLUT‐4 expression. In addition, molecules such as the Akt substrate of 160 kDa (AS160), which mediate GLUT‐4 translocation to the cell membrane, are reduced in endometrial IR, further compromising endometrial receptivity (ER) [60]. Expression levels of components in the mammalian target of rapamycin (mTOR) signalling pathway are closely linked to ER. In maternal hyperinsulinemia mouse models, the expression of phosphorylated mTOR (p‐mTOR) and phosphorylated p70S6 kinase (p‐p70S6K) is decreased, accompanied by dysregulation of ER‐related genes such as Esr1, Pgr, Hoxa10, and Esr2. Notably, insulin treatment was able to restore ER function and normalise gene expression, indicating that local endometrial IR directly affects the transcriptional regulation of ER‐associated genes [59].

#### 3.2.2 Influence the Pathway of Glucose Uptake by Endometrium

One of the key features of local insulin resistance (IR) in the endometrium is impairment of the insulin receptor substrate (IRS)‐phosphoinositide 3‐kinase (PI3K)‐protein kinase B (Akt) signalling pathway. The PI3K/Akt cascade is central to endometrial glucose uptake and plays a critical role in regulating cellular proliferation, survival, and differentiation [61]. Most insulin signal transduction is mediated through this pathway, with Akt acting as the central effector. This pathway facilitates the translocation of glucose transporters (GLUTs) to the cell membrane, enabling glucose uptake and supporting various cellular processes. In patients with IR, elevated insulin levels modulate apoptosis via the PI3K/phosphorylated‐Akt (p‐Akt) axis, leading to a significant reduction in the expression of decidual markers such as bone morphogenetic protein 2 (BMP2), oestrogen receptor (ER), and progesterone receptor (PR) at embryo implantation sites and within the endometrium. Moreover, this dysregulation compromises uterine stromal cell function and increases mitochondrial membrane potential, thereby impairing decidualisation in early pregnancy models [62]. Currently, it is widely accepted that the primary mechanism of IR involves disruptions in signal transduction downstream of insulin receptor binding. Aberrations in molecules such as IRS, Akt, and GLUTs are considered major contributors to local endometrial IR in patients with polycystic ovary syndrome (PCOS) [63]. Such impairments compromise endometrial receptivity and are a critical cause of infertility.

### 3.3 Infertility Caused by IR via Destroyed Endometrial Receptivity

Studies have demonstrated that under conditions of hyperinsulinemia and elevated blood glucose, the expression levels of decidualisation markers, such as bone morphogenetic protein 2 (BMP2) and prolactin (PRL), are significantly downregulated. Furthermore, vascular endothelial growth factor A (VEGFA), a key regulator of endometrial angiogenesis, is markedly reduced both in vivo and in vitro, suggesting that hyperinsulinemia impairs endometrial vascular remodelling during early pregnancy in mice [64]. As discussed previously, local insulin resistance (IR) disrupts the expression of molecules associated with endometrial receptivity and key components of the glucose metabolic pathway, resulting in glucose deficiency within endometrial tissue. Such metabolic disturbances compromise endometrial proliferation and differentiation, leading to failed embryo implantation or an elevated risk of pregnancy loss. Meanwhile, in vitro studies have indicated that cortisol impairs endometrial insulin sensitivity, reduces glucose uptake by endometrial cells, and interferes with endometrial receptivity, implying that the significantly decreased pregnancy rate in patients with IR is primarily attributed to impaired endometrial function and defective embryo attachment [65].

## 4 IR Affects Endometrial Receptivity via Regulating Glucose Metabolism

### 4.1 Background

Endometrial glucose metabolism involves several tightly regulated steps. Glucose is primarily transported via glucose transporter 4 (GLUT4), facilitating its diffusion across the epithelium into the uterine cavity or its conversion into fructose and lactate through the polyol pathway or glycolysis, followed by secretion into the uterine lumen. Once glucose enters epithelial cells, it is stored as glycogen and subsequently mobilised via glycogenolysis. Decidualisation significantly alters glucose metabolism in the endometrial stroma. During this process, glucose flux through the pentose phosphate pathway increases to support nucleic acid synthesis and NADPH production. Post‐decidualisation, cells shift toward a metabolism dominated by aerobic glycolysis, with reduced activity of the tricarboxylic acid (TCA) cycle and oxidative phosphorylation (OXPHOS). Additionally, decidualisation is accompanied by extensive glycosylation of extracellular proteins, including N‐glycan and O‐linked β‐N‐acetylglucosamine (O‐GlcNAc) modifications. The decidua also accumulates substantial glycogen reserves to support early pregnancy demands [66].

### 4.2 The IRS‐PI3K‐Akt‐GLUTs Pathway (Figure 2)

**FIGURE 2:**
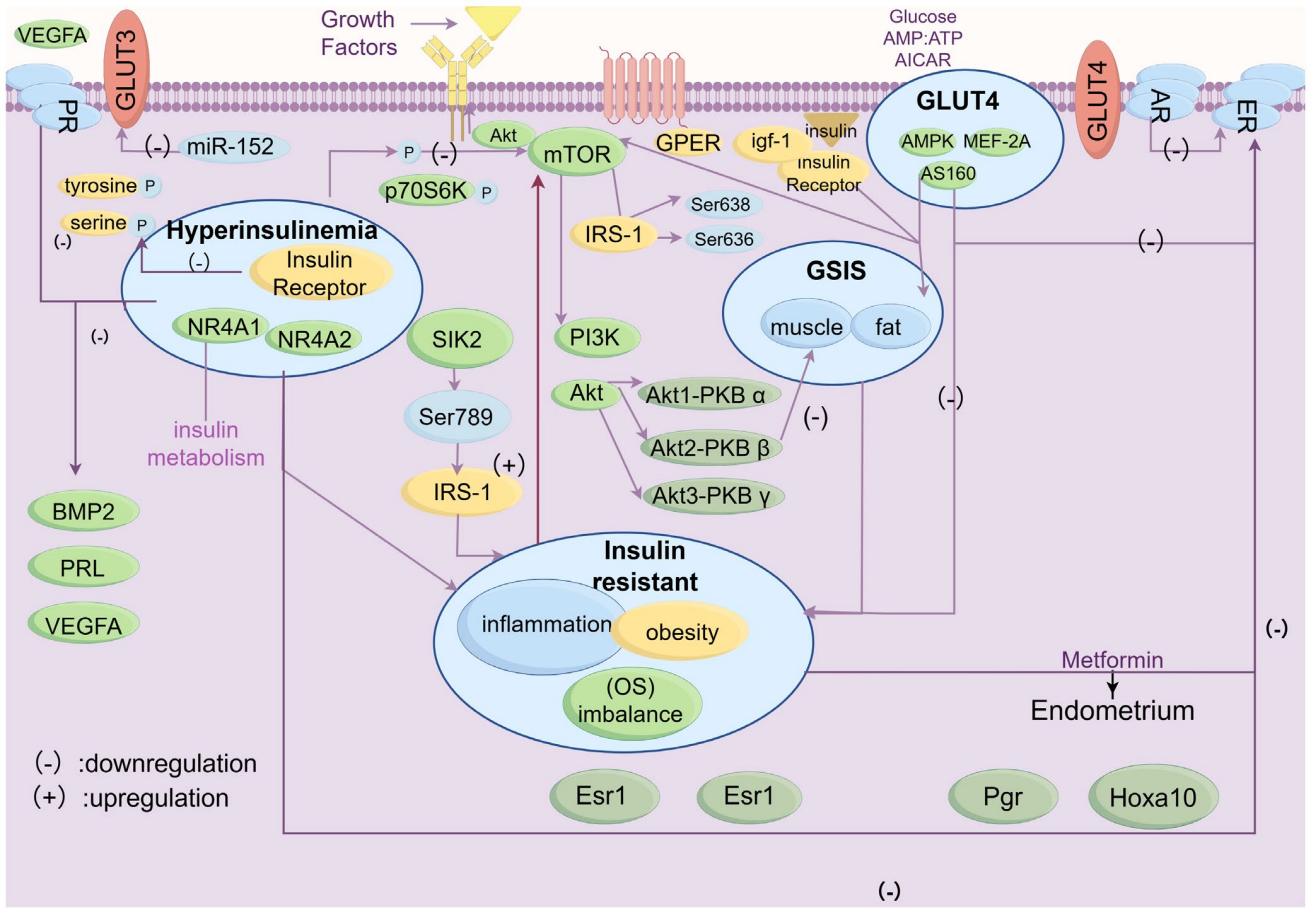
The mechanisms of insulin resistance in the endometrium.

Schematic representation of the molecular mechanisms involved in insulin resistance, hyperinsulinaemia, and GSIS. The diagram illustrates the role of insulin signalling pathways, including the activation and inhibition of key molecules such as IRS‐1, PI3K, Akt, and mTOR. The regulation of GLUT4 translocation and glucose uptake is also depicted, with specific emphasis on the impact of growth factors, inflammatory responses, and metabolic imbalance. Downregulation (−) and upregulation (+) of key molecular factors are indicated. The effects of metformin on endometrial function and the involvement of steroid hormone receptors (PR, AR, ER) are also highlighted.

#### 4.2.1 IRS

Insulin receptor substrates 1 and 2 (IRS1/IRS2) are essential mediators in the early stages of insulin signalling and are impaired in endometrial insulin resistance (IR). Immunohistochemical analysis of endometrial tissue from polycystic ovary syndrome (PCOS) patients with normoinsulinemia (PCOSE‐NI) and hyperinsulinemia (PCOSE‐HI) demonstrated no significant differences in insulin receptor (INSR) expression among groups. However, Western blot analysis revealed significantly reduced IRS1 expression in the PCOSE‐HI group compared to the PCOSE‐NI group [67]. Although IRS1 is critical for transmitting insulin signals, its overexpression or abnormal serine phosphorylation may contribute to the development of IR. For example, salt‐inducible kinase 2 (SIK2), a member of the AMP‐activated protein kinase (AMPK) family, can phosphorylate IRS1 at Ser789, impairing its function. Similarly, the mammalian target of rapamycin (mTOR) pathway modulates IRS1 activity through phosphorylation at Ser636/639, further disrupting downstream insulin signalling [68].

#### 4.2.2 PI3K‐Akt

Insulin can directly promote cell proliferation via the PI3K/Akt signalling pathway. Under insulin resistance (IR), impairment of this pathway leads to reduced expression of key components, thereby affecting endometrial cell proliferation and differentiation, ultimately diminishing endometrial receptivity. Increasing evidence suggests that various pharmacological agents can alleviate IR by modulating the PI3K/Akt pathway [63]. Akt, also known as protein kinase B (PKB), is a cytoplasmic serine/threonine kinase essential for cell survival and proliferation, both of which are critical for successful implantation. There are three isoforms of Akt: Akt1 (PKBα), Akt2 (PKBβ), and Akt3 (PKBγ), each playing distinct roles in metabolic and physiological regulation. Akt1 deficiency in mice results in reduced cell survival and increased perinatal mortality. Loss of Akt2 impairs insulin‐stimulated glucose uptake in skeletal muscle and adipose tissue, leading to systemic insulin intolerance. Although Akt3 deficiency does not affect glucose metabolism, it leads to a marked reduction in brain volume, total cell number, and average cell size. All three isoforms are activated by PI3K and mediate growth factor signalling [69].

#### 4.2.3 GLUTs

Of the 14 known GLUTs, 9 have been detected in the uterus (Table 2, especially in GLUT4). To date, 14 glucose transporter (GLUT) isoforms have been identified, of which nine have been detected in the uterus. This section details their localisation, expression levels, and functional significance, as summarised in Table 2 [66].

**TABLE 2:** The localisation, level of expression, and representative significance of common glucose transporters in the endometrium.

GLUT type	Location	Level of expression	Representative meaning
GLUT1	All cell types of the endometrium contain	The endometrial epithelial cell content is greater than the stromal cell content. It increases tenfold during decidualisation.	During the process of decidualisation, glucose uptake increases accordingly. During this period, abnormal glucose metabolism can affect the amount of GLUT1, leading to a decrease in endometrial receptivity.
GLUT3	High‐glucose‐requiring tissues	Constant levels are expressed in the proliferative phase of menstruation or early pregnancy and in the decidua. It is expressed in the trophoblast in early pregnancy and is not expressed in the placenta. A slight decrease is observed in the second trimester.	Decline in the second trimester
GLUT4	Endometrial epithelial cells and endometrial stromal cells	Expression is elevated during the follicular phase GLUT4 is significantly decreased in hyperinsulinemia During the menstrual cycle, P4 induces the expression of GLUT4 in the endometrium	PCOS can disrupt the P4‐induced GLUT4 expression pathway. New therapeutic targets can be found by studying this pathway.

GLUT1, the first identified in the endometrium, is present in all three major endometrial cell types. In the non‐pregnant uterus, SLC2A1 (GLUT1) expression is higher in epithelial cells than in stromal cells [27]. During pregnancy, GLUT1 levels are markedly elevated in the decidua. Decidualisation induces a tenfold increase in GLUT1 protein, accompanied by a corresponding rise in glucose uptake. During this process, progesterone (P4) upregulates GLUT1, while 17β‐estradiol (E2) downregulates it, suggesting a possible antagonistic interaction. In vivo GLUT1 knockout leads to implantation failure, underscoring the critical role of glucose metabolism in endometrial receptivity [23, 26].

GLUT3, typically expressed in tissues with high glucose demand, is stably expressed during the proliferative phase and in the decidua during early pregnancy. In early gestation, GLUT3 is restricted to trophoblasts and is absent in term placenta. Its expression slightly declines in the second trimester, indicating reduced involvement at later stages. Moreover, GLUT3 expression in the uterus and placenta correlates with serum progesterone levels during early pregnancy [24].

GLUT4, a key insulin‐responsive transporter, shows low expression in the endometrium [24, 26]. Although current data on endometrial GLUT4 expression are scarce and inconsistent, its importance in the endometrium is widely recognised. It is localised to both epithelial and stromal cells [70], with significantly higher mRNA expression during the follicular phase and a decrease in the luteal phase [71]. Compared with PCOS patients without insulin resistance (IR), endometrial GLUT4 levels are markedly lower in both hyperinsulinemic and obese PCOS patients, suggesting that hyperinsulinemia and obesity contribute to GLUT4 downregulation, which has also been confirmed by in vivo studies [72]. Insulin signals GLUT4 translocation in insulin‐sensitive tissues, including the endometrial stroma, via insulin and IGF‐I receptors. Dysregulated GLUT4 expression may alter the metabolic and hormonal milieu in hyperinsulinemic PCOS. Although P4 modulates endometrial GLUT4 expression throughout the menstrual cycle, this regulation is disrupted in PCOS, impairing normal GLUT4 dynamics [25]. A deeper understanding of insulin signalling and GLUT4 regulation in the endometrium may provide valuable insights into the pathophysiology of PCOS and offer potential therapeutic targets [67].

### 4.3 The cAMP‐PKA‐Ca ^2+^ Pathway

Follicle‐stimulating hormone (FSH) exerts its reproductive function by binding to the FSH receptor (FSHR) and activating the cAMP–PKA signalling pathway [73]. Beyond its classical role, FSH also influences other tissues. Recent studies have detected FSHR expression in pancreatic islet β‐cells in both humans and mice, where FSH regulates glucose‐stimulated insulin secretion (GSIS) in a bell‐shaped dose–response curve. Mechanistically, FSH primarily signals through FSHR by activating Gαs, thereby stimulating the cAMP/PKA and calcium signalling pathways to enhance GSIS. However, at higher concentrations, FSH activates Gαi, which suppresses the cAMP/PKA pathway and intensifies its regulatory effects on insulin secretion [19]. Moreover, FSH concentrations below 10 IU/L modulate intracellular cAMP levels in a dose‐dependent manner. Concurrently, FSH promotes calcium influx from extracellular sources via membrane‐bound Ca^2+^channels, forming a complete transcriptional cascade that contributes to β‐cell function.

## 5 New Direction of Treatment: Preclinical and Clinical Examples (Figure 3)

**FIGURE 3:**
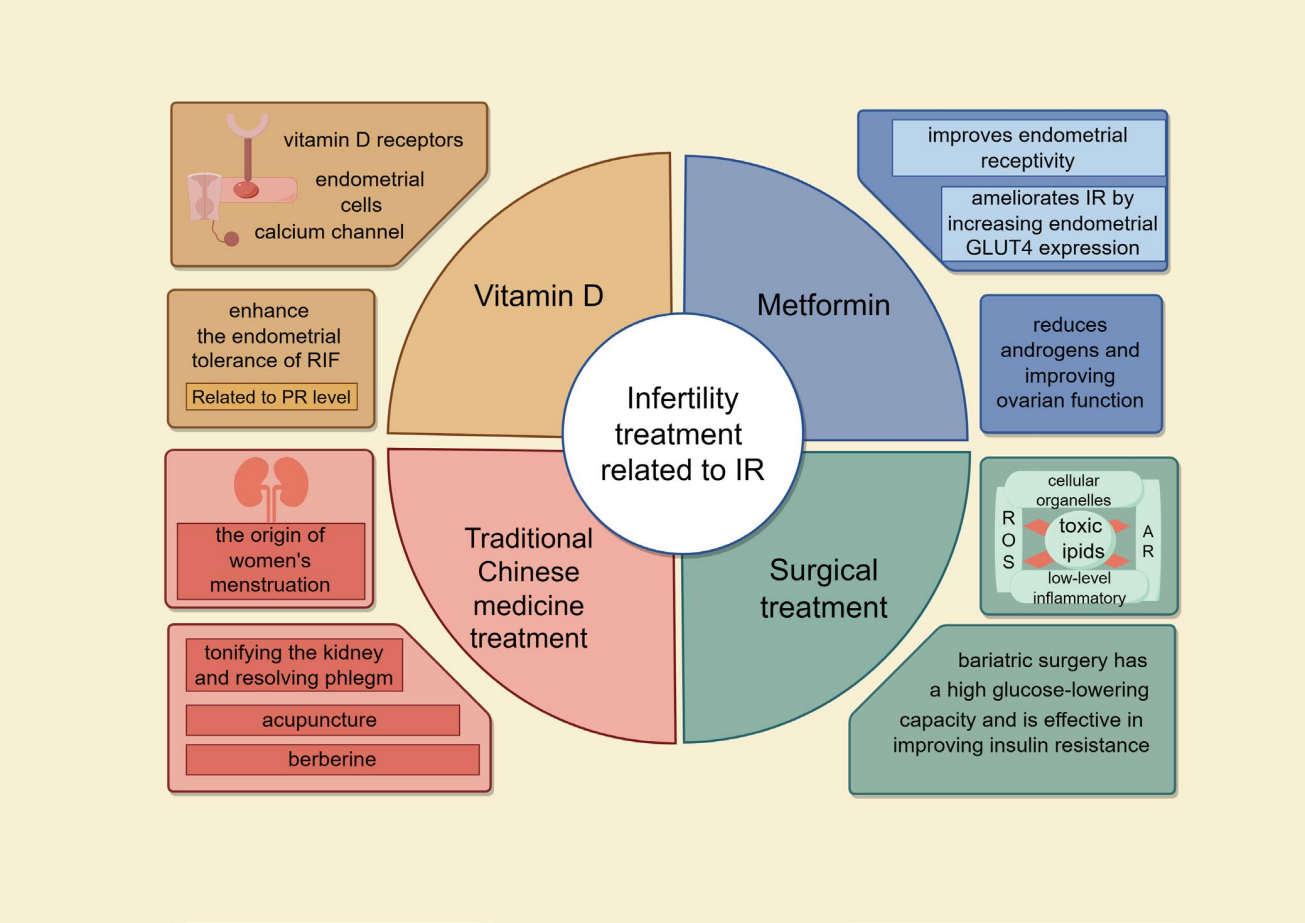
Infertility treatment related to IR.

This figure outlines various infertility treatments linked to IR. It highlights how Vitamin D, Metformin, TCM, and surgical interventions contribute to improving fertility outcomes. Vitamin D enhances endometrial receptivity and insulin sensitivity through its receptors and calcium channels in endometrial cells. Metformin ameliorates IR by boosting endometrial GLUT4 expression and reducing androgens, thereby improving ovarian function. TCM approaches, such as kidney‐tonifying and phlegm‐resolving therapies, acupuncture, and berberine use, address the root of women's menstrual issues. Surgical treatments, particularly bariatric surgery, effectively lower glucose levels and enhance insulin sensitivity. The figure encapsulates the diverse mechanisms and benefits of these treatments in managing infertility associated with insulin resistance.

### 5.1 Pharmacological Treatments

#### 5.1.1 Vitamin D

Recent studies have indicated that co‐administration of vitamin D and metformin provides superior therapeutic outcomes compared to metformin alone. Emerging evidence supports a critical role of vitamin D deficiency in the pathogenesis of metabolic dysfunction and infertility in women with polycystic ovary syndrome (PCOS). For optimal reproductive outcomes, low‐dose vitamin D supplementation may be most effective when administered to selected populations at specific stages of the ovarian cycle [74, 75]. The presence of vitamin D receptors in the endometrium suggests a regulatory role in endometrial receptivity via both calcium‐dependent and independent signalling pathways [76]. Preliminary prospective studies have demonstrated a potential association between vitamin D supplementation and improved IVF implantation rates, although larger, well‐designed trials are warranted to validate these findings [76]. Furthermore, in patients with recurrent implantation failure (RIF), vitamin D has been shown to enhance endometrial receptivity by modulating progesterone receptor (PR) expression. Specifically, vitamin D increases PR mRNA and protein levels, as well as phosphorylation at Ser294, in isolated endometrial stromal cells (eSCs) from both RIF patients and healthy controls [77, 78].

#### 5.1.2 Metformin

Metformin, a biguanide and first‐line therapeutic agent for type 2 diabetes mellitus, reduces insulin resistance and inhibits hepatic gluconeogenesis by enhancing insulin sensitivity [79]. Several studies have demonstrated that metformin improves endometrial receptivity, potentially by upregulating endometrial receptivity markers such as HOXA10 and leukaemia inhibitory factor (LIF) [80]. A reliable meta‐analysis provides compelling evidence that metformin enhances endometrial receptivity by increasing endometrial thickness (EMT) and reducing endometrial arterial resistance index (RI). Further studies are warranted to evaluate molecular markers of receptivity, alongside large‐scale, multicentre, randomised controlled trials [81]. In PCOS mouse models, metformin treatment was associated with increased EMT and myometrial thickness, alongside a reduction in the length of normal glands and blood vessels, suggesting a possible correlation with localised endometrial insulin resistance (IR). Consistent findings from three‐dimensional power Doppler ultrasonography (3DPDUS) in patients treated with metformin for 6 months indicated a significant increase in both endometrial thickness and volume [82]. Moreover, all parameters related to uterine vascularisation were significantly improved in metformin‐treated PCOS patients [83]. Related evidence suggests that early intervention with liraglutide, a GLUT1 agonist, may enhance fertility potential by more than doubling IVF pregnancy rates compared to metformin. However, its direct effect on endometrial receptivity remains to be elucidated. Further investigation is required to clarify the role of GLUT1 in endometrial physiology, especially in relation to previous findings on GLUT1 expression [84]. Mechanistically, metformin enhances endometrial insulin sensitivity by upregulating GLUT4 expression through the PI3K‐Akt and AMPK‐Rab4 signalling pathways. Rab4, activated by metformin, facilitates the translocation of GLUT4 to the plasma membrane, highlighting the significance of metformin's direct action on endometrial cells [85]. Additionally, metformin can act synergistically with other treatments. For example, its combination with a carbohydrate‐controlled diet has been shown to improve endometrial function partly via modulation of DNA methylation at the HOXA10 gene promoter [86]. Beyond its endometrial effects, metformin also improves ovarian function and reduces androgen levels, which is particularly beneficial in the treatment of PCOS. Targeting localised endometrial IR may represent a novel therapeutic approach for PCOS‐associated infertility. Current evidence suggests that insulin‐sensitising pharmacologic strategies, including metformin, could play a central role in future infertility treatments for patients with PCOS [87].

### 5.2 Surgical Treatment: Bariatric Surgery

As previously discussed, obesity is closely associated with insulin resistance. Adipose tissue accumulation leads to the release of lipotoxic substances, resulting in organelle dysfunction, elevated levels of reactive oxygen species (ROS), and a chronic low‐grade inflammatory state, ultimately disrupting glucose homeostasis [88]. A significant proportion of infertile women with polycystic ovary syndrome (PCOS) are obese, suggesting a potential link between obesity and infertility [89]. Multiple mechanisms have been proposed to explain this relationship. First, elevated levels of toxic lipids, leptin, and free fatty acids (FFA) may impair endometrial function and reduce endometrial receptivity. Second, obesity contributes to peripheral insulin resistance and enhances androgen production, leading to functional hyperandrogenism and impaired fertility [90]. However, the extent to which obesity‐related endometrial dysfunction accounts for infertility in women remains unclear and warrants further investigation [91]. From a metabolic perspective, bariatric surgery exerts potent glucose‐lowering effects and significantly improves insulin sensitivity. Given the role of insulin resistance in endometrial dysfunction and infertility, bariatric surgery presents a promising therapeutic approach for infertile women with obesity and PCOS [92].

### 5.3 Traditional Chinese Medicine Treatment

In Traditional Chinese Medicine (TCM), menstruation is believed to originate from kidney function. Deficiency in kidney essence may lead to irregular or absent menstruation. Infertility in obese women is traditionally attributed to uterine obstruction caused by excessive adipose tissue, with treatment strategies focused on resolving phlegm and tonifying the kidneys. Based on this theory, TCM recommends the use of kidney‐tonifying and phlegm‐resolving therapies for infertile patients with PCOS and obesity [93]. Recent evidence supports the efficacy of acupuncture in improving reproductive outcomes in PCOS. In animal models, acupuncture was shown to promote endometrial angiogenesis by activating the PI3K‐Akt signalling pathway, aligning with the TCM concept of replenishing qi and nourishing the blood [94]. From a metabolic standpoint, berberine—a major bioactive compound derived from Coptis chinensis—has demonstrated the ability to ameliorate insulin resistance in murine models. This effect is mediated, at least in part, by activation of the GLP‐1/GLP‐1R/PKA signalling cascade [95]. Given the role of insulin resistance in PCOS‐associated infertility, berberine may offer therapeutic potential. Emerging clinical studies have shown that berberine improves fertility and pregnancy outcomes in affected individuals [96]. Mechanistically, berberine has been found to enhance ovulatory function in PCOS by upregulating luteinising hormone/choriogonadotropin receptor (LHCGR) and cytochrome P450 family 19 subfamily A member 1 (CYP19A1). Additionally, it improves endometrial receptivity by downregulating integrin αvβ3 and lysophosphatidic acid receptor 3 (LPAR3), further supporting its role in fertility management [97].

## 6 Conclusion and Prospective

This review integrates the physiological and pathological aspects of endometrial glucose metabolism, particularly in the context of insulin resistance. It highlights the roles of hormonal regulation, glucose transporters (GLUTs), and glycolytic pathways, and mechanistically elucidates how local insulin resistance impairs endometrial receptivity. Central pathways such as IRS–PI3K–Akt and cAMP–PKA are emphasised for their involvement in this process. Under normal physiological conditions, glucose metabolism ensures adequate energy supply for the endometrium during the ‘implantation window’, thus supporting successful implantation. However, in states of insulin resistance, hyperinsulinaemia disrupts glucose homeostasis, impairs endometrial function, and contributes to implantation failure and subsequent infertility. Studies have indicated that patients with polycystic ovary syndrome (PCOS) undergoing assisted reproductive technology (ART) exhibit low embryo transfer success rates, which are closely associated with insulin resistance and hyperinsulinaemia. For instance, a clinical trial conducted by Daimin Wei et al. in PCOS patients confirmed that preconception impaired glucose tolerance (IGT), independent of body mass index (BMI), is associated with adverse pregnancy outcomes compared with individuals with isolated impaired fasting glucose (i‐IFG) and those with normoglycemia [98].

For long‐term therapeutic strategies, it is crucial to identify key metabolic targets associated with glucose utilisation. Interventions during the window of implantation should focus on molecules closely related to endometrial receptivity, including GLUT1, GLUT4, FOXO1, FSH, and HOXA10. Targeted modulation of these factors may improve embryo implantation. This suggests that future controlled clinical studies could categorise patients into recurrent embryo transfer and non‐recurrent embryo transfer groups to compare endometrial GLUT expression, as well as to evaluate clinical outcomes such as embryo implantation rate and pregnancy rate, thereby establishing the association between GLUTs and embryo implantation.

Moreover, future research should aim to establish reliable biomarkers for the evaluation of endometrial receptivity, which remains a significant gap in clinical diagnostics and treatment monitoring. Proteomic analyses have demonstrated that metformin modulates the expression of several endometrium‐associated proteins, including IGFBP‐7, integrin αvβ3, LIF, apolipoprotein D, angiomodulin, and PYGB. Among these, the upregulation of IGFBP‐7 is the most prominent, suggesting its potential as a biomarker for endometrial receptivity. Nevertheless, its clinical utility requires further validation in expanded studies, and we hypothesise that it may be correlated with implantation outcomes [99]. To date, a number of interventional studies have evaluated the effects of metabolic therapy on endometrial receptivity. For example, Yugang Chi et al. reported that following transcervical adhesiolysis, patients with intrauterine adhesions exhibited significantly increased expression of the endometrial receptivity markers αvβ3 and laminin, which enhanced endometrial receptivity and helped improve reproductive prognosis in these patients [100]. Accordingly, assessing the impact of metabolic therapy on endometrial receptivity holds considerable clinical promise.

## 7 Search Strategy and the Inclusion/Exclusion Criteria

### 7.1 Inclusion Criteria

Literature screening was performed based on keywords, abstracts, figures, tables, and conclusions, with the following specific requirements:

1. Search strategy: Keywords ‘Endometrial receptivity’, ‘glucose metabolism’, and ‘insulin resistance’ were searched in PubMed.
2. Publication time: Literature and studies published within the recent decade.
3. Research objective: Studies investigating the influencing factors and mechanisms underlying endometrial receptivity and glucose metabolism under insulin resistance.
4. Study type: Mechanistic studies, animal experiments, cell experiments, clinical studies, and reviews (selected according to the research objective).
5. Outcome: Studies are consistent with the conclusion that insulin resistance affects endometrial receptivity.

### 7.2 Exclusion Criteria

1. Duplicate publications were removed after importing literature into EndNote.
2. Literature published more than 10 years ago.
3. Literature irrelevant to the research objective and outcome measures.
4. Literature with serious data missing, inappropriate statistical methods, uninterpretable results, duplicate publication, or redundant submission.
5. Case reports, commentaries, expert consensuses, editorials, and abstract‐only publications.

## Author Contributions

**Shao‐rong Chen:** writing – review and editing, project administration, supervision, investigation, validation, conceptualization, visualization. **Qi‐rong Shi:** methodology, supervision, resources, writing – review and editing. **Ling‐tao Zheng:** writing – review and editing, supervision, methodology, conceptualization, investigation. **Si‐yan Huang:** writing – original draft, writing – review and editing, conceptualization, methodology, investigation, formal analysis, visualization. **Shu Lin:** project administration, formal analysis, supervision, writing – review and editing. **Jing Xu:** conceptualization, writing – review and editing, methodology, supervision, visualization. **Wei‐hong Chen:** conceptualization, supervision, visualization, resources, writing – review and editing. **Yan‐Chuan Shi:** conceptualization, methodology, writing – review and editing, investigation, supervision, project administration.

## Funding

This work was supported by the Science and technology project of Fujian Provincial Health Commission (grant number 2020CXB027,2022CXA038), the Youth Research Project of Fujian Provincial Health Commission (No. 2022QNA067), Fujian Province Science and Technology Innovation Joint Fund Project (Grant number 2023Y9254).

## Conflicts of Interest

The authors declare no conflicts of interest.

## Data Availability

Data sharing not applicable to this article as no datasets were generated or analysed during the current study.
